# Association between Overweight and Diet Diversity Score: A Cross-Sectional Study Conducted among Tunisian Children

**DOI:** 10.3390/children8070536

**Published:** 2021-06-24

**Authors:** Darine Dogui, Radhouene Doggui, Jalila El Ati, Myriam El Ati-Hellal

**Affiliations:** 1 INNTA (National Institute of Nutrition and Food Technology), SURVEN (Nutrition Surveillance and Epidemiology in Tunisia) Research Laboratory, Tunis 1007, Tunisia; deryn_dogui@yahoo.fr (D.D.); jalila.elati@yahoo.fr (J.E.A.); 2 INAT (National Agronomic Institute of Tunisia), University of Carthage, Tunis 1082, Tunisia; 3 Centre de Formation Médicale du Nouveau-Brunswick (Université de Sherbrooke), Pavillon J.-Raymond-Frenette, Université de Moncton, 18 Antonine-Maillet Ave, Moncton, NB E1A 3E9, Canada; 4 Department of Family and Emergency Medicine, Université de Sherbrooke, Sherbrooke, QC J1K 2R1, Canada; 5 IPEST (Preparatory Institute for Scientific and Technical Studies), University of Carthage, Laboratory Materials Molecules and Applications, P.B. 51, Tunis 2070, Tunisia; mfh22002@yahoo.fr

**Keywords:** diet quality, nutrients, obesity, childhood, Tunisia, low- and middle-income countries

## Abstract

Aim: This study explored the association between the diet diversity score (DDS) and overweight among Tunisian children. Methods: A representative sample of children living in Greater Tunis was selected based on a two-stage clustered sampling design. A total of 1200 children (3–9 years) were recruited. Dietary assessment was realized using a 24 h dietary recall. Anthropometric measurements were realized, and overweight was defined according to the World Health Organization standards. Logistic regression was used for the association between DDS with overweight. Results: A quarter of children were found to be overweight. Overweight prevalence was found to decrease with the increase of mother education level (*p* = 0.010) among children <6 years. Crude DDS score was higher among non-overweight children irrespective of the age class (*p* = 0.002). Tunisian children appeared to consume much more than six food groups, corresponding to a more than recommended intake of most nutrients. Intriguingly, DDS was positively associated with the occurrence of overweight children <6 years, adjusted odd ratio = 1.37, 95% CI (1.03–1.82). Conclusion: Overweight is a public health problem among Tunisian children. A high DDS signifies adequate nutrient intake. An increase of DDS was found to be a positive predictor of overweight only in pre-school children.

## 1. Introduction

Obesity constitutes a global public health concern, as it affected 107.7 million (98.7–118.4) children during 2015. Globally, the death attributed to obesity was found to reach an alarming rate of four million people [1]. In Tunisia, the nutritional transition has been documented among adults as a trigger and sustainable cause for the emergence of overweight and obesity [2,3,4]. According to non-communicable diseases (NCDs) workgroup estimates, the overweight prevalence among Tunisian children and adolescents shifted from 3.2% in 1973 to 24% in 2016 for boys and from 6.2% in 1973 to 25.9% in 2016 for girls, respectively [5]. A regional study found that overweight and obesity prevail among 9.1% and 11.6% of pre-school children, respectively [6]. 

Malnutrition is considered as an early life adversity, and its effect could last to adulthood [7]. Therefore, diet quality (including the nutrients intake) is crucial for children’s physical and mental growth [8]. A meta-analysis conducted by *Liberali* et al. [9] mapped dietary patterns associated with an increased risk of obesity and found that children (aged 1 to 6 years) adhering to ‘snack’, ‘westernized’ and ‘traditional’ diets are more likely to be obese. 

Several nutritional tools were developed because of the complexities of characterizing dietary quality [10]. Among them the “a priori” approach, which is evidence-based [11,12] and both provides diet diversity scores (DDS) [13]. Irrespective of the constructs and cut-offs used to build the score, the DDS score has a multidimensional aspect, and as such, is a proxy indicator of nutrient adequacy [14], and an increased score mirrors a high diet quality and adequate nutrient intake [15,16]. The DDS is computed from a number of specific food groups with a minimum of four groups. The DDS is a useful tool predicting nutrient intake adequacy, especially in low- and middle-income countries. The DDS increase has been negatively associated with chronic conditions, e.g., diabetes [17] and metabolic syndrome [18]. Nonetheless, at the time of the study, conflicting results are merging as regards its association with overweight and obesity among adolescents and adults [19]. Because obesity has its root during the early years of life, and dietary patterns tend to track from childhood to adulthood, there is an increasing importance in assessing the risk factors of childhood obesity. Scarce data are available on the association of food variety and weight status among school and pre-school children. A conducted study among children in the United States found that the DDS is positively associated with a greater body mass for index z-score [20]. Another Malaysian study found that higher DDS is positively associated with weight for the age z score among children aged 1 to 6 years [21]. However, the association between food variety and weight status is not unanimous [22].

The following study assessed: (*i*) the diet diversity of Tunisian children aged from 3 to 9 y; (*ii*) the prevalence of overweight and associated factors; (*iii*) the relationship between overweight and the diet diversity among the studied children.

## 2. Methodology

### 2.1. Population

The following study was conducted from April to May 2017 in Greater Tunis, the most developed region in the country as well as the most urban, which encompasses four governorates (*Tunis*, *Manouba*, *Ariana* and *Ben Arous*). The subjects were selected based on a two-stage stratified clustered sampling design carried out by the National Institute of Statistics. At the first level, 30 primary schools and 30 kindergartens were randomly selected from the initial sampling frame. At the second level, 20 children were systematically drawn from each educational institution. A total of 1200 children were recruited for the following study aged between 3 and 9 years.

The calculation of the sample size was done using the following:

$$n=t^{2}\times p\times \left(1-p\right)\times DEFF\times \frac{1}{d^{2}}$$

where *n* = sample size, *t* = constant (2.045 for *df* = 29 and *p* = 0.05), *p* = expected prevalence (18.4%), *d* = relative desired precision (fixed at 0.3) and the Designed effect (*DEFF*) = 1.7

### 2.2. Dietary Intake Assessment

Trained dietitians conducted face-to-face 24-h dietary recalls with a parent of each child during household visits or at the school after scheduling a meeting. Through a face-to-face interview, the dieticians recorded all foods and beverages that the child consumed the previous day. The amount of food and number of beverages were estimated using household measures or food photographs. In addition, the dieticians recorded the diet history for the month preceding the survey. On the day of the survey, unclear descriptions or doubtful records were checked with the parents of the child. To increase the accuracy of the records during the interview, the dieticians used the Tunisian guide for portion size of foods using photos of food portions and specific portions [23]. The energy and nutritional content of identified food items and recipes were estimated by laboratory analysis, the Tunisian food composition table [24], The US Department of Agriculture table [25] and food processor software [26]. The revised version of the Association of Official Agricultural Chemists official method 996.06 was adopted for total fat, saturated fatty acids and trans-fatty acids analysis [27]. The 24 h dietary recall was validated against the three-day food record method among school-age children (6 to 12 years) [28]. The adequacy of intake was assessed using the recommended dietary reference intakes of the French population [29].

### 2.3. Diet Diversity Score

The DDS was based on ten food groups according to the Food and Agriculture Organization recommendation. The defined food groups are: ‘*All starch staples*’, ‘*vitamin A—rich vegetables and fruits*’, ‘*All other fruits*’, ‘*All other vegetables*’, ‘*All legumes and nuts*’, ‘*oil and fat*’, ‘*Meat, poultry and fish*’, ‘*All dairy*’, ‘*Eggs*’ and ‘*other foods*’ (not retained for the calculation of the DDS score) [30]. A cut-off of 10 g was applied for the whole food groups except for oil and fat (2 g [31]), so for example, if a given child reaches the intake of 10 g for the ‘egg’ group, they receive one point. The total DDS score is the sum of the different subgroups’ scores. All subgroups have the same weight and the score ranges from 0 to 9.

### 2.4. Anthropometric Measures

Standing height was measured to the nearest 0.1 cm with the use of a wall-mounted stadiometer (Person-check^®^, Kirchner and Wilhelm, Asperg, Germany); weight was measured to the nearest 0.1 kg with a calibrated scale (Detecto, Webb City, MO, USA). BMI (Body Mass Index = weight/height^2^) for-age z-scores were derived from the World Health Organization (WHO) reference for school-age children and overweight (including obesity) was defined as BMI-for-age ≥ +1 z [32]. For children under 5 years, overweight was defined as BMI-for-age ≥ +2 z [33,34,35].

### 2.5. Demographic and Socio-Economic Characteristics

Data on the level of education and occupation of children’s parents as well as the living area were collected using an auto-administered questionnaire, sent a few days before the survey, and returned by the children the day of the survey.

A household economic score was computed by multiple correspondence analyses from six variables describing the characteristics of the dwelling and eleven variables coding household ownership of appliances [36]. The score was divided into tertiles that express ‘high’, ‘medium’ or ‘low’ household economic level.

### 2.6. Data Analysis

Epidata 3.1 software was used for the data entry. To minimize errors, two different agents oversaw the procedure. To account for the complex survey design, the STATA 16.1 *svy* function was used. Continuous variables were expressed as weighted mean ± standard error of mean and categorical variables as a weighted percentage with 95% confidence interval (CI). The mean comparison was carried out by linear regression, while proportion comparison was done by the Chi-squared test. The binary logistic regression was used (i.e., healthy weight vs. overweight/obese) to examine the association with the main independent variable (DDS score) and co-variable (sex, parents’ education level, household head professional status and household economic level) of adjustment. The Wald test was used for the regression coefficient comparison [37]. Finally, Student’s t-test was used for the comparison of mean values to a reference value (measured coverage for nutrient intakes vs. the 100% as a reference value). The probability level was set at 0.05.

## 3. Results

### 3.1. Demographics

A total of 532 children (279 boys and 253 girls) aged 3–5 years and 632 children (303 boys and 329 girls) aged 6–9 years were surveyed, from which dietary, socio-demographic and anthropometric data were collected (Table 1). The non-participation rate was 3%, which refers to the participants that were absent on the day of the survey. Most fathers and mothers had secondary schooling or more, but among children under 6 years, parents appeared to be higher educated (*p* from <0.0001 to 0.0005). The head of the household for more than half of the children worked as an employee and one-third of them as a middle or upper executive. Consistently, parents of children under 6 years had a significantly higher percentage of household heads that worked as a middle executive (*p* = 0.022). Finally, a higher household economic level was found among pre-school children (*p* = 0.012). 

### 3.2. Overweight Prevalence by Socio-Economic Characteristics

The prevalence of overweight (including obesity) seems to increase with age (*p* = 0.0007). Table 2 shows the distribution of overweight according to the socio-economic characteristics by age. For the younger age group (<6 years), low mother education level was associated with the higher prevalence of overweight. Conversely, an increase in overweight was associated with the father’s education level (no formal education, 17.8% vs. university level, 37.6%; *p* = 0.042) for the older group (≥6 years). Consistently, overweight was associated with an increase in household economic level (low, 18.5% vs. high, 36.0%; *p* = 0.0003).

### 3.3. Analysis of Energy and Nutrient Intakes

Table 3 shows the average intakes of energy and nutrients among the assessed children. Overall, energy and nutrient intakes were more frequently higher among children aged 6 years old and above. However, sugars, vitamin A—RAE, vitamin B5, vitamin B6, vitamin B12, potassium and calcium intakes were significantly higher among children aged under 6 years. Considering the whole sample, the contribution of carbohydrates was as high as 58.1%, while the contribution of fat was around 29.6%. No substantial difference was found for the contribution of fats and carbohydrates to the daily energy intake between the two age groups. The contribution of sugar to the daily energy intake exceeded 15% and was significantly higher among children under 6 years (*p* < 0.0001). 

**Figure 1:**
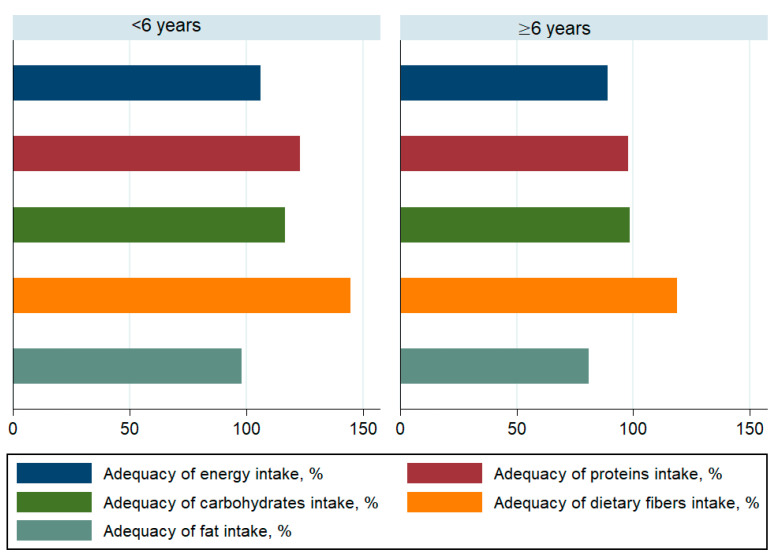
Adequacy of macronutrient intake among children by age.

**Figure 2:**
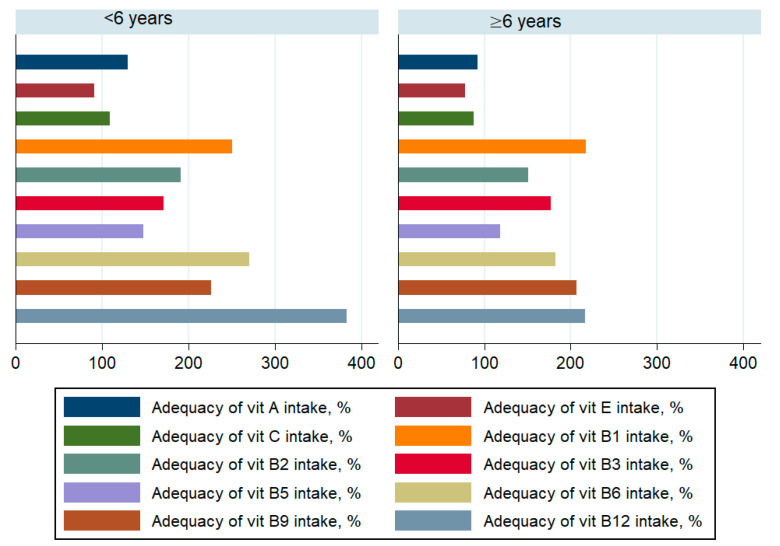
Adequacy of vitamin intake among children by age.

**Figure 3:**
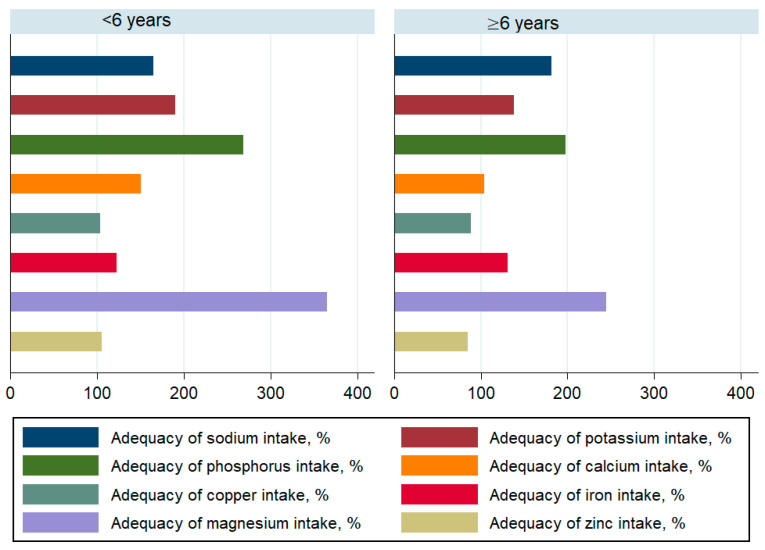
Adequacy of mineral and trace element intake among children by age.

The daily contribution of saturated fatty acids to the energy intake was higher than 10% in both groups. Children under 6 years old achieved adequate coverage for energy, macronutrients (Figure 1), vitamins-except vitamin E-(Figure 2) and minerals (Figure 3). The intakes of children ≥ 6 years did not reach the optimal coverage for energy, proteins, fat intake, vitamin A, vitamin E, vitamin C and zinc.

### 3.4. Diet Diversity Score by Age and Overweight Status

Table 4 shows the distribution of DDS and its subscores according to the overweight status. Overall, the common observation between the two age groups is that the DDS score and its subscores are significantly higher in non-overweight children. Some exceptions were raised from the analysis where the non-significant difference was found for the ‘*all other fruits*’, ‘*all legumes and nuts*’ and ‘*eggs*’ subscores among children under 6 years. For the older group, the ‘all other vegetables’ subscore approached significance (*p* = 0.061). Additionally, no significant trend was depicted for the ‘all other fruits’, ‘oil and fat’ and ‘eggs’ subscores.

### 3.5. Individual-Level Association between Overweight Status, Diet Diversity Score and Socio-Economic Characteristics

*Children < 6 years.* In crude analysis (Table 5), the increase of DDS score was found to be a protective factor against overweight (crude OR = 0.81, 95% C.I. (0.72–0.89). In adjusted analysis, the increase of the DDS score by one unit was associated with a 1.4-time increase of the overweight risk. The increase of the mother education level, at least primary schooling level vs. no formal schooling, was found to be a protective factor against overweight (adjusted OR = 0.12, 95% C.I. (0.01–0.85).

*Children ≥ 6 years.* In crude analysis (Table 5), the increase of the DDS was found to be a protective factor against overweight (OR = 0.84, 95% CI: (0.77–0.91)). In adjusted analysis, this association did not stand. The increase of household economic level was associated with an increased overweight likelihood irrespective of the adjustment level.

## 4. Discussion

This is the first Tunisian study assessing the diet diversity among Tunisian children in association with overweight. We found that the diet diversity score was positively associated with the occurrence of overweight pre-school children. A high prevalence of overweight (including obesity) was found, which seems to increase with age. Tunisian children showed an acceptable DDS when they eat more than six food groups per day on average. 

The high prevalence of overweight complies with the nutrition transition in Tunisia during the last decades. In 1997, a national survey showed that overweight prevalence among pre-school and school-age children was less than 5% [38]. Another regional study among school-age children (*n* = 1569) reported that overweight affected 11.6% and 16.1% of boys and girls, respectively [39]. Similar findings of a positive trend regarding the excess of adiposity have been reported elsewhere [40]. The progress of overweight is a public health concern in Tunisia and other North African countries. Nowadays, children are exposed to a more obesogenic environment, as they tend to consume more processed and ready-to-eat food with a high content of added sugar and fat [41]. An analysis of the most consumed industrial products by children as well as fast food revealed a high amount of trans-fatty acids [41,42]. Moreover, while no official assessment of food advertisements has been conducted in Tunisia, similar countries from the Eastern and Mediterranean regions are frequently exposed to unhealthy food advertisements in mass media [43]. The anthropometric parameters trends in Tunisia are similar to those reported in proximal North African countries (e.g., Algeria and Morocco) which showed a rising of the excess of adiposity [44,45,46,47].

Our study showed that energy and macronutrient intakes were sufficient among pre-school and comparison groups. While fat intake fell significantly below 100% of coverage among children 6 years of age and older, it is probably because the used reference of intake is not specific to the Tunisian population and may introduce some bias. Vitamin intake profile analysis showed an acceptable coverage to a very high intake, especially for the B vitamins. A very high intake of phosphorus, sodium, potassium and magnesium was found among minerals and trace elements. While the reasonable higher intake of potassium may moderate the high intake of sodium [48], the latter could be responsible for the early onset of chronic diseases, such as hypertension. This, in turn, could increase the likelihood of chronic disease that occur during adulthood.

Our analysis showed that one unit’s increase of the DDS is associated with 1.4 more risks of being overweight among pre-school children. The diet diversity approach probably ignores some important contributors to the daily energy intake, i.e., sugary and sweetened beverages. Moreover, the DDS does not mirror to which extent the intakes of a certain food are high. For example, in Tunisia, bread consumption is very high, and it is documented to be 245 g per day among adults [49]. In Tunisia, starch staples contribute to a non-neglect part of the daily energy intake, which could increase the likelihood of overweight and obesity [50]. Additionally, the increase of food variety in the diet could be associated with an increase of body fat [51]. An increase in food diversity has been correlated with higher energy intake [16].

Our analysis also showed that the increase of the mother’s education level is a strong and positive predictor of the DDS. This highlights the importance of including the promotion of a healthy diet among mothers in policies aiming to tackle overweight and obesity. Finally, the overweight likelihood was found to increase in association with the economic level. Probably, children living in a household with a high income have more access to food, and their daily energy intake might be higher [52].

### Limitations

This study has limitations; indeed, it was conducted in one of the most developed regions in Tunisia. Nevertheless, it can be generalized to regions nearby that have reached the same level of development at the country or the East Mediterranean region level. The use of a 24 h dietary recall could introduce intra-individual bias. However, the importance of the sample size could partially overcome this issue. The lack of seasonality information as well as the level of physical activity could be considered important confounders in our study. 

## 5. Conclusions

The high prevalence of overweight among Tunisian children confirms the nutrition transition. An increased DDS appeared to comply with a context of sufficient intake of energy and nutrients. However, this score does not consider the amount of food consumed and the daily energy intake, which may explain the intriguing positive association with overweight. Reconsidering the overall efficacy of DDS as a measure of diet quality and building other validated DDS tools to be used are highly recommended.

## Figures and Tables

**Table 1:** Socio-demographic characteristics of 3–9-year-old children (*n* = 1164).

	*n*	Age Group		*p*-Value
		<6 Years	≥6 Years	
Sex, female, %		51.8 ^a^	47.9	0.19
Father education level, %				
No formal education	10	0.3	0.9	0.0005
Primary schooling	268	18.1	27.9	
Secondary schooling	463	40.7	39.2	
University level	409	40.5	32.0	
Mother education level, %				
No formal education	33	1.5	3.9	<0.0001
Primary schooling	250	14.2	27.7	
Secondary schooling	421	35.2	36.9	
University level	455	49.1	31.5	
Household head occupation				
Not working	15	1.2	1.3	0.022
Worker/employee	674	55.1	62.3	
Middle executive	211	22.6	15.8	
Upper executive	230	21.1	20.3	
Household economic level				
Low	366	28.8	36.7	0.012
Medium	375	37.4	30.8	
High	362	33.7	32.5	

^a^ Weighted percentage.

**Table 2:** Distribution of overweight (including obesity) among Tunisian children by age.

	Age Group			
	<6 Years		≥6 Years	
	%	95% C.I.	%	95% C.I.
Sex, %	*p*^a^ =0.54			
	*p*^b^ = 0.99		*p*^c^ = 0.99	
Boys	21.7	17.2–27.0	29.3	24.4–34.7
Girls	21.7	17.0–27.3	31.8	27.4–37.1
Father education level, %	*p* = 0.39			
	*p* = 0.19		*p* = 0.042	
No formal education	47.6	10.9–87.1	17.8	2.4–65.3
Primary schooling	18.5	11.9–27.7	24.4	18.6–31.3
Secondary schooling	25.2	19.8–31.4	30.2	24.8–36.3
University level	18.6	13.8–24.6	37.6	31.2–44.5
Mother education level, %	*p* = 0.28			
	*p* = 0.010		*p* = 0.15	
No formal education	67.0	31.8–89.9	23.2	10.7–24.5
Primary schooling	21.4	13.6–31.9	24.5	18.7–31.5
Secondary schooling	23.8	18.2–30.4	32.4	26.6–38.7
University level	18.8	14.4–24.1	34.3	28.0–41.2
Household head occupation, %	*p* = 0.99			
	*p* = 0.49		*p* = 0.31	
Not working	31.9	8.3–70.9	12.7	1.8–54.4
Worker/employee	22.9	18.4–28.2	29.0	24.7–33.8
Middle executive	21.0	14.4–29.7	32.7	24.2–42.7
Upper executive	16.4	10.4–24.8	36.0	28.0–44.8
Household economic level, %	*p* = 0.038			
	*p* = 0.73		*p* = 0.0003	
Low	18.5	13.1–25.6	18.5	13.9–24.2
Medium	20.6	15.3–27.1	30.6	24.3–37.6
High	17.3	12.2–24.0	36.0	29.6–43.1

^a^ Chi squared test *p*-value for the overall sample. ^b^ Chi squared test for the children under 6 years. ^c^ Chi squared test *p*-value for the children age 6 years and older.

**Table 3:** Distributions of energy and nutrient intakes by age groups.

	Total	<6 Years	≥6 Years	*p*-Value
Energy (kcal)	1510 ± 7.9 ^a^	1446.3 ± 10.7	1581.0 ± 11.0	<0.0001
Proteins (g/d)	55.3 ± 0.4	54.1 ± 0.7	56.6 ± 0.6	0.005
Energy from proteins (%)	14.8 ± 0.1	15.1 ± 0.2	14.4 ± 0.1	0.002
Carbohydrates (g/d)	218.9 ± 1.4	208.7 ± 1.9	230.0 ± 1.9	<0.0001
Energy from carbohydrates (%)	58.1 ± 0.3	58.0 ± 0.5	58.3 ± 0.3	0.62
Total sugar (g/d)	86.6 ± 1.0	91.9 ± 1.4	90.9 ± 1.4	<0.0001
Energy from sugars (%)	23.1 ± 0.3	25.5 ± 0.4	20.4 ± 0.3	<0.0001
Dietary fibers (g/d)	14.8 ± 0.2	14.1 ± 0.3	15.4 ± 0.2	<0.0001
Fats (g/d)	49.7 ± 0.5	47.8 ± 0.7	51.7 ± 0.6	<0.0001
Energy from fats (%)	29.6 ± 0.3	29.8 ± 0.6	29.3 ± 0.3	0.42
SFA ^b^ (mg/d)	19.2 ± 0.2	18.8 ± 0.3	19.7 ± 0.3	0.058
Energy from SFA (%)	11.4 ± 0.1	11.6 ± 0.2	11.1 ± 0.1	0.008
MUFA ^c^ (mg/d)	17.2 ± 0.2	16.4 ± 0.3	18.1 ± 0.2	<0.0001
Energy from MUFA (%)	10.2 ± 0.1	10.2 ± 0.1	10.3 ± 0.1	0.46
PUFA ^d^ (mg/d)	11.4 ± 0.2	10.6 ± 0.2	12.2 ± 0.2	<0.0001
Energy from PUFA (%)	6.78 ± 0.08	6.6 ± 0.1	7.0 ± 0.1	0.008
Vitamin A—RAE ^e^ (µg/d)	511.2 ± 25.8	566.2 ± 47.2	451.1 ± 15.4	0.021
Vitamin E (alpha tocopherol) mg/d	6.54 ± 0.15	6.4 ± 0.2	6.7 ± 0.2	0.36
Vitamin C (mg/d)	76.4 ± 2.3	77.6 ± 3.4	75.1 ± 3.1	0.57
Vitamin B1 (mg/d)	1.47 ± 0.02	1.34 ± 0.02	1.61 ± 0.02	<0.0001
Vitamin B2 (mg/d)	1.80 ± 0.02	1.80 ± 0.04	1.82 ± 0.02	0.54
Vitamin B3 (mg/d)	14.1 ± 0.2	12.7 ± 0.2	15.5 ± 0.2	<0.0001
Vitamin B5 (mg/d)	4.11 ± 0.04	4.22 ± 0.07	3.99 ± 0.04	0.005
Vitamin B6 (mg/d)	1.86 ± 0.03	2.00 ± 0.05	1.72 ± 0.04	<0.0001
Vitamin B9 (mg/d)	342.0 ± 4.4	304.3 ± 5.7	382.9 ± 6.2	<0.0001
Vitamin B12 (µg/d)	3.34 ± 0.29	3.81 ± 0.53	2.86 ± 0.15	0.089
Sodium (mg/d)	2087 ± 19	1889 ± 24	2304 ± 25	<0.0001
Potassium (mg/d)	1944 ± 17	2022 ± 25	1859 ± 23	<0.0001
Phosphorus (mg/d)	1115 ± 8	1134 ± 12	1095 ± 11	0.018
Calcium (mg/d)	916.9 ± 8.2	955.0 ± 13.0	875.3 ± 12.1	<0.0001
Copper (mg/d)	1.00 ± 0.03	0.97 ± 0.05	1.01 ± 0.02	0.47
Iron (mg/d)	9.32 ± 0.09	8.55 ± 0.13	10.2 ± 0.10	<0.0001
Magnesium (mg/d)	422.9 ± 4.2	411.5 ± 5.9	435.4 ± 5.9	0.004
Zinc (mg/d)	7.11 ± 0.06	7.10 ± 0.08	7.13 ± 0.07	0.74

^a^ Weighted mean ± standard error of mean. ^b^ Saturated fatty acids. ^c^ Monounsaturated fatty acids. ^d^ Polyunsaturated fatty acids. ^e^ Retinol acid equivalent.

**Table 4:** Distribution of the diet diversity score and subscores by overweight (including obesity) status and age.

	<6 years (*n* = 532)			≥6 years (*n* = 632)		
	Non-Overweight	Overweight	*p*-Value	Non-Overweight	Overweight	*p*-Value
DDS score	6.87 ± 0.07 ^a^	6.04 ± 0.26	0.002	6.82 ± 0.07	6.21 ± 0.18	0.002
All starch staples score	0.98 ± 0.01	0.83 ± 0.03	<0.001	0.98 ± 0.01	0.87 ± 0.02	<0.0001
Vitamin A-rich vegetables and fruit score	0.89 ± 0.02	0.77 ± 0.04	0.004	0.91 ± 0.01	0.83 ± 0.02	0.014
All other fruits score	0.24 ± 0.02	0.24 ± 0.04	0.90	0.19 ± 0.02	0.17 ± 0.03	0.44
All other vegetables score	0.86 ± 0.02	0.77 ± 0.04	0.023	0.86 ± 0.02	0.80 ± 0.03	0.061
All legumes and nuts score	0.80 ± 0.02	0.71 ± 0.04	0.052	0.78 ± 0.02	0.68 ± 0.03	0.013
Oil and fat score	0.81 ± 0.02	0.72 ± 0.04	0.034	0.82 ± 0.02	0.79 ± 0.03	0.38
Meat, poultry, fish score	0.96 ± 0.01	0.81 ± 0.03	<0.0001	0.96 ± 0.01	0.87 ± 0.02	<0.0001
All dairy score	0.98 ± 0.01	0.82 ± 0.03	<0.0001	0.93 ± 0.01	0.82 ± 0.03	<0.0001
Eggs score	0.33 ± 0.02	0.37 ± 0.04	0.56	0.36 ± 0.02	0.36 ± 0.03	0.95

^a^ weighted mean ± standard error of mean.

**Table 5:** Logistic regression for the subject level association between overweight (including obesity) and diet diversity score.

	<6 Years				≥6 Years			
	Crude Analysis		Adjusted Analysis		Crude Analysis		Adjusted Analysis	
	OR ^a^	95% C.I. ^b^	OR ^a^	95% C.I. ^b^	OR ^a^	95% C.I. ^b^	OR ^a^	95% C.I. ^b^
	*p*^c^ < 0.0001		*p*^c^ = 0.030		*p*^c^ < 0.0001		*p*^c^ = 0.093	
DDS	0.81	0.72–0.89	1.37	1.03–1.82	0.84	0.77–0.91	1.15	0.97–1.35
Sex	*p*^c^ = 0.99		*p*^c^ = 0.74		*p*^c^ = 0.48		*p*^c^ = 0.27	
Boys	1		1		1		1	
Girls	1.00	0.65–1.52	1.08	0.67–1.75	1.12	0.80–1.58	1.23	0.84–1.79
Father education level	*p*^c^ = 0.21		*p*^c^ = 0.47		*p*^c^ = 0.045		*p*^c^ = 0.74	
No formal education	1		1		1		1	
Primary schooling	0.25	0.03–2.00	0.49	0.04–5.52	1.49	0.16–13.29	0.95	0.10–8.96
Secondary schooling	0.37	0.05–2.81	0.97	0.10–9.68	2.00	0.22–17.69	0.99	0.10–9.20
University level	0.25	0.03–1.92	0.95	0.10–10.5	2.78	0.32–24.7	1.39	0.13–13.91
Mother education level	*p*^c^ = 0.033		*p*^c^ = 0.084		*p*^c^ = 0.15		*p*^c^ = 0.62	
No formal education	1		1		1		1	
Primary schooling	0.13	0.02–0.64	0.12	0.01–0.85	1.08	0.40–2.89	1.26	0.37–4.22
Secondary schooling	0.15	0.03–0.69	0.10	0.02–0.62	1.58	0.60–4.15	1.31	0.39–4.36
University level	0.11	0.02–0.51	0.09	0.01–0.57	1.72	0.65–4.55	0.93	0.26–3.31
Household head occupation	*p*^c^ = 0.50		*p*^c^ = 0.42		*p*^c^ = 0.34		*p*^c^ = 0.96	
Not working	1		1		1		1	
Worker/employee	0.63	0.12–3.37	0.36	0.07–2.00	2.80	0.33–23.3	1.51	0.19–11.71
Middle executive	0.56	0.10–3.14	0.35	0.06–2.16	3.33	0.39–28.5	1.34	0.15–11.41
Upper executive	0.42	0.07–2.34	0.22	0.03–1.51	3.85	0.45–32.55	1.37	0.16–11.50
Household economic level	*p*^c^ = 0.73		*p*^c^ = 0.54		*p*^c^ = 0.0004		*p*^c^ = 0.030	
Low	1		1		1		1	
Medium	1.13	0.65–1.97	1.45	0.74–2.85	1.93	1.21–3.08	1.81	1.04–3.15
High	0.91	0.51–1.64	1.31	0.59–2.89	2.47	1.58–3.88	2.30	1.22–4.33

^a^ Crude or adjusted odds ratio. ^b^ 0.95 sampling design-based confidence interval for crude or adjusted odds ratio. ^c^ Crude or adjusted *p*-value for the association of overweight with co-factor.

## Data Availability

The data are available upon reasonable request from the corresponding author.
